# Sarcopenia and myosteatosis diagnostic tool for gastrointestinal cancer: creatinine to cystatin C ratio as evaluation marker

**DOI:** 10.1186/s12967-023-04628-z

**Published:** 2023-10-20

**Authors:** Hao Liu, Junjie Wang, Shanjun Tan, Zhige Zhang, Mingyue Yan, Jun Han, Xiangyu Sui, Fan Yang, Guohao Wu

**Affiliations:** Department of General Surgery/Shanghai Clinical Nutrition Research Center, Zhongshan Hospital, Fudan University, 180 Fenglin Road, Xuhui District, Shanghai, 200032 China

**Keywords:** Sarcopenia, Myosteatosis, Creatinine to cystatin C ratio, Diagnostic tool

## Abstract

**Objective:**

This study aimed to develop a simplified diagnostic tool for assessing sarcopenia and myosteatosis in gastrointestinal cancer patients, focusing on the creatinine to cystatin C ratio (CCR) as an evaluation marker.

**Methods:**

955 patients were split into training (n = 671) and validation (n = 284) cohorts. Using logistic regression, risk factors for sarcopenia and myosteatosis were identified. The predictive capacity of the developed model was examined. The association between CCR and muscle imaging parameters, along with its impact on clinical outcomes, was analyzed.

**Results:**

No significant differences were observed in baseline traits between cohorts. CCR emerged as a significant risk factor for both sarcopenia and myosteatosis. Nomograms for diagnosing these conditions demonstrated strong predictive ability, with AUC values indicating high accuracy (sarcopenia AUC: 0.865–0.872; myosteatosis AUC: 0.848–0.849). The clinical utility of the nomograms was confirmed through decision curve analysis. CCR showed significant association with muscle imaging parameters and was a reliable indicator for assessing the risk of sarcopenia, myosteatosis, and cachexia. Moreover, CCR was able to differentiate between patient survival and disease progression rates.

**Conclusion:**

A diagnostic tool for sarcopenia and myosteatosis in gastrointestinal cancer patients was developed, with CCR being a pivotal biomarker for disease diagnosis and prognosis prediction.

**Graphical Abstract:**

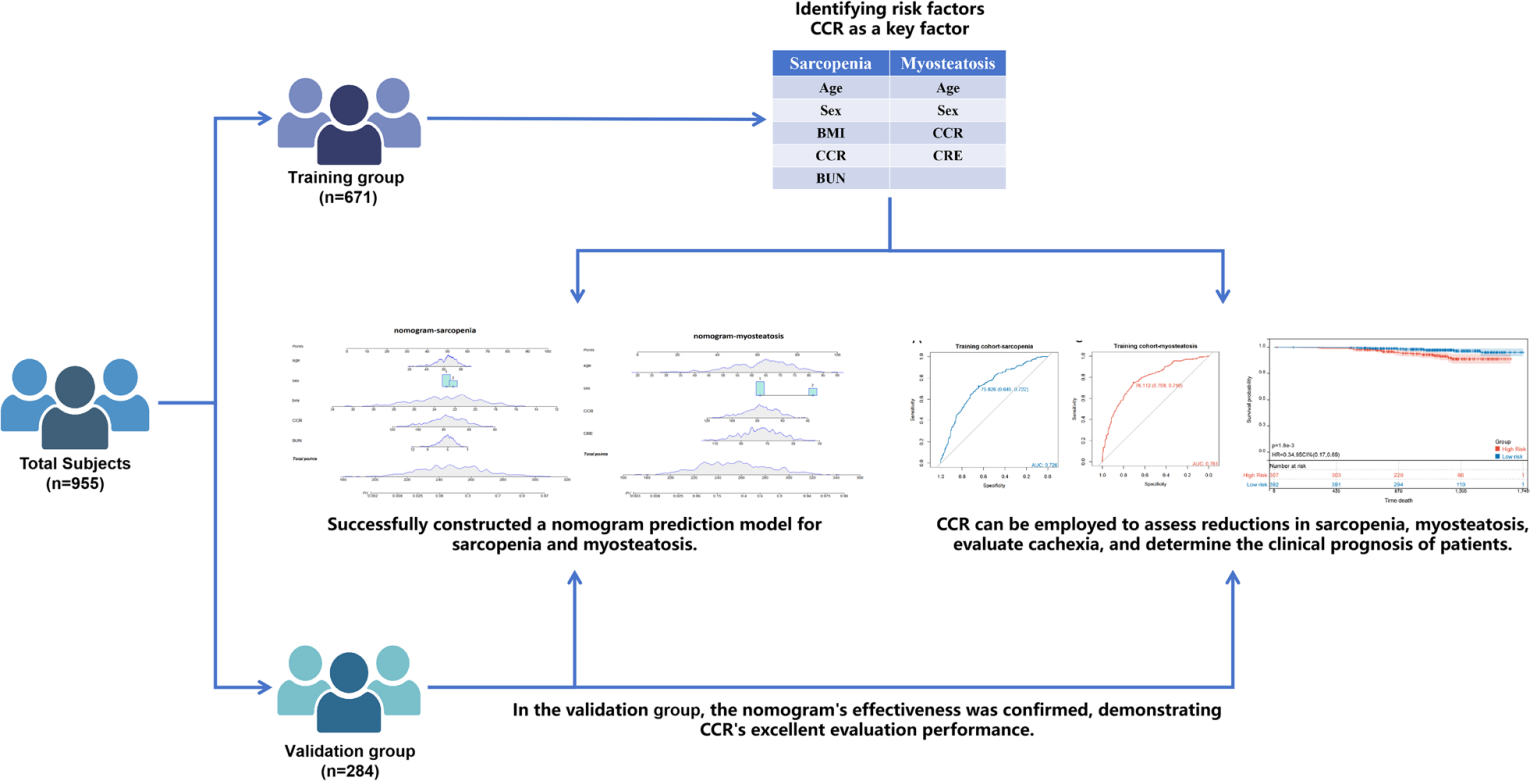

**Supplementary Information:**

The online version contains supplementary material available at 10.1186/s12967-023-04628-z.

## Background

Sarcopenia refers to the loss of muscle strength and mass, and its presence is associated with poorer health outcomes in all populations [1, 2]. Muscle fat infiltration, or myosteatosis, is a condition in which there is an increase in fat infiltration in the muscles, leading to a decrease in overall strength and function [3, 4]. Research has shown that sarcopenia and myosteatosis are key factors affecting patient treatment efficacy, functional status, and quality of life [5, 6]. Cachexia is a systemic wasting syndrome caused by chronic serious illness, characterized by muscle loss [7]. Muscle atrophy and myosteatosis occur before the onset of cancer cachexia and lead to its development [8, 9]. Early diagnosis and timely intervention of muscle atrophy and myosteatosis can improve the condition of patients and prevent complications.

Currently, various methods are used in clinical practice to detect sarcopenia and myosteatosis, such as CT, MRI, and DXA [10, 11]. However, due to their high cost and time-consuming nature, they are not universally applicable in clinical settings. Therefore, identifying serum biomarkers that can be used to assess skeletal muscle-related parameters in patients is crucial for clinical diagnosis and evaluation.

Serum creatinine and cystatin C are commonly used biomarkers for evaluating kidney function in clinical practice [12]. CRE varies with body composition, while cystatin C is widely present in nucleated cells and is less affected by muscle mass [13]. Therefore, the serum creatinine-to-cystatin C ratio (CCR) may be a promising alternative biomarker for sarcopenia.

Therefore, this study aims to thoroughly evaluate the CCR as a potential indicator for diagnosing and assessing muscle atrophy and myosteatosis. Additionally, we further seek to develop a diagnostic model based on CCR for skeletal muscle reduction syndrome and musculoskeletal disorders in gastrointestinal cancer patients.

## Method

### Study population

The study population of this research is patients with gastrointestinal tumors. The research data is sourced from our clinical database, covering the time period from January 2018 to December 2023. Inclusion criteria: age greater than 18 years. Exclusion criteria: patients with a pathological diagnosis of benign diseases, acute kidney injury, missing abdominal CT images, low-quality CT images or patients with any anatomical deformities (such as abdominal wall edema), and a history of previous abdominal surgery. This study has been approved by the hospital ethics committee and informed consent has been obtained from all patients.

### Data collection

We collected demographic and clinical data, including the following variables: age, gender, height, weight, body mass index (BMI), tumor location, tumor necrosis factor (TNF), C-reactive protein (CRP), glucose (GLU), albumin (ALB), neutrophils (NEUT), lymphocytes (LYMPH), hemoglobin (HGB), white blood cells (WBC), platelets (PLT), serum creatinine (CRE), serum cystatin C (CYSC), creatinine to cystatin C ratio (CCR = CRE/CYSC), and blood urea nitrogen (BUN).

Imaging indicators include: skeletal muscle index (SMI), skeletal muscle density (SMD), skeletal muscle weight (SMG), skeletal muscle area (SMA), intermuscular adipose tissue percentage (IMAT%), and intermuscular adipose tissue (IMAT) cross-sectional area.

We also collected follow-up information, including patients' mortality, disease progression, time of death, and time of disease progression.

Blood collection standards require patients to fast for more than 8 h and collect blood from the patient’s cubital vein in the morning to measure biochemical indicators.The BMI calculation formula is: BMI = weight (kg)/height^2^ (m^2^).

### Diagnostic criteria

This study used SMI to adjust for the impact of body size on evaluating skeletal muscle mass. SMI was calculated as SMI = SMA (cm^2^)/height squared (m^2^), with higher SMI indicating more skeletal muscle. Based on our previous research, the cut-off values for male muscle atrophy were 43.13 cm^2^/m^2^ and for female 37.81 cm^2^/m^2^ [14]. Higher area percent of intermuscular adipose tissue (IMAT) indicates worse muscle mass [15]. We used IMAT% as an indicator of muscle quality, calculated as IMAT% = IMAT/SMA × 100%. IMAT% > 7.51% for males or > 6.83% for females was considered as muscle fat infiltration [16]. The diagnostic criteria for cachexia were based on previous international consensus statements [7]: (1) Weight loss > 5% in the past 6 months without dieting; (2) BMI < 20 kg/m^2^ and weight loss > 2%; (3) decrease in skeletal muscle mass and sustained weight loss > 2%.

### Construction of a forest plot and performance evaluation methods

We randomly divided the study subjects into a training group and a validation group in a 7:3 ratio. Independent prognostic factors were screened from the training set using univariate and multivariate logistic regression analysis and then used to construct a forest plot, calculate the hazard ratio (HR) and 95% confidence interval (CI). The predictive performance of the model was evaluated using the area under the curve (AUC). The similarity between the predicted and actual results was compared using calibration curves. Clinical utility and net benefit were evaluated using decision curve analysis [17].

### Correlation analysis and risk survival analysis

The correlation between CCR and different radiological indicators was evaluated using a scatter plot with Spearman’s rank correlation coefficient. The ability of CCR to evaluate muscle atrophy and muscle fat infiltration was evaluated using AUC, and the optimal cut-off value was determined. The relationship between CCR and clinical outcomes was analyzed using Kaplan–Meier curves.

### Statistical analysis

Frequency and mean ± standard deviation (SD) of baseline data were used to describe the characteristics of the two groups of patients. Continuous variables were analyzed using *t*-tests, and categorical variables were analyzed using Fisher’s exact test. All statistical analyses were performed using SPSS software version 26.0 (IBM SPSS Inc., New York, USA) and R version 4.2.1 (R Foundation for Statistical Computing, Vienna, Austria). P-values less than 0.05 were considered statistically significant.

## Results

### Baseline patient characteristics

Table 1 shows the baseline characteristics of the two patient groups, and randomization results indicated no significant differences between the groups. In assessing sarcopenia, single-factor and multi-factor logistic regression analyses found age, sex, BMI, CCR, and BUN to be significant variables and independent predictors of sarcopenia (Additional file 1: Table S1). In assessing myosteatosis, single-factor and multi-factor logistic regression analyses found age, sex, CCR, and CRE to be significant variables and independent predictors of myosteatosis (Additional file 2: Table S2).

**Table 1:** Patient characteristics

Characteristic	Overall (955)	Training cohort (671)	Validation cohort (284)	p-value
Basic information				
Age (year)	63.07 (10.89)	62.89 (10.98)	63.50 (10.71)	0.424
Sex (n)				0.881
Male	630 (66%)	444 (66%)	186 (65%)	
Female	325 (34%)	227 (34%)	98 (35%)	
Weight(kg)	62.93 (10.72)	62.53 (10.84)	63.88 (10.39)	0.071
Height(m)	1.65 (0.08)	1.65 (0.08)	1.65 (0.08)	0.883
BMI(kg/m^2^)	23.00 (3.20)	22.84 (3.22)	23.37 (3.12)	0.018
BSA	1.69 (0.17)	1.69 (0.17)	1.71 (0.17)	0.125
Location(n)				0.724
Colorectum	483 (51%)	342 (51%)	141 (50%)	
Stomach	472 (49%)	329 (49%)	143 (50%)	
Ratio of body weight loss	0.02 (0.05)	0.02 (0.05)	0.02 (0.05)	0.933
CCR(umol/mg)	77.79 (13.23)	77.91 (13.12)	77.52 (13.51)	0.688
Cre(umol/L)	75.66 (14.98)	75.58 (14.96)	75.85 (15.05)	0.797
CysC(mg/L)	0.98 (0.17)	0.98 (0.16)	0.99 (0.17)	0.387
TNF(ng/ml)	17.04 (23.12)	16.73 (21.93)	17.78 (25.74)	0.549
CRP(mg/L)	6.24 (15.87)	5.97 (15.12)	6.88 (17.53)	0.448
BUN(mmol/L)	5.24 (1.52)	5.26 (1.48)	5.18 (1.62)	0.490
GLU(mmol/L)	5.58 (1.47)	5.57 (1.48)	5.59 (1.45)	0.905
ALB(g/L)	41.08 (4.27)	41.13 (4.30)	40.95 (4.20)	0.558
NEUT(× 10^9^/L)	3.39 (1.61)	3.36 (1.66)	3.47 (1.48)	0.331
LYMPH(× 10^9^/L)	1.66 (0.57)	1.67 (0.56)	1.64 (0.57)	0.347
HGB(× 10^12^/L)	122.64 (23.49)	122.71 (23.18)	122.47 (24.26)	0.888
WBC(× 10^9^/L)	5.72 (1.93)	5.69 (1.98)	5.78 (1.82)	0.516
PLT(× 10^9^/L)	234.38 (80.98)	234.14 (79.79)	234.94 (83.88)	0.892
Imaging indices				
SMI(cm^2^/m^2^)	46.49 (7.70)	46.35 (7.50)	46.80 (8.14)	0.423
IMAT%	6.84 (5.54)	6.54 (3.96)	0.08 (0.08)	0.047
IMAT(cm^2^)	7.98 (4.51)	7.84 (4.31)	8.33 (4.93)	0.146
SMD (HU)	32.94 (6.30)	33.16 (6.17)	32.42 (6.60)	0.109
SMA(cm^2^)	127.68 (26.41)	127.31 (26.04)	128.55 (27.29)	0.517
SMG(AU)	1548.76 (446.56)	1552.87 (434.00)	1539.04 (475.57)	0.674
Clinical outcomes				
Cachexia(n)				0.531
No	771 (81%)	538 (80%)	233 (82%)	
Yes	184 (19%)	133 (20%)	51 (18%)	
Sarcopenia(n)				0.627
No	710 (74%)	502 (75%)	208 (73%)	
Yes	245 (26%)	169 (25%)	76 (27%)	
Myosteatosis(n)				0.080
No	596 (62%)	431 (64%)	165 (58%)	
Yes	359 (38%)	240 (36%)	119 (42%)	
Death(n)				0.507
No	663 (95%)	476 (95%)	187 (94%)	
Yes	36 (5%)	24 (5%)	12 (6%)	
Missing	256	171	85	
Progression(n)				0.878
No	615 (89%)	435 (90%)	180 (90%)	
Yes	73 (11%)	51 (10%)	22 (10%)	
Missing	267	185	82	
OS(day)				0.565
	1110.37 (298.77)	1114.47 (299.79)	1100.09 (296.72)	
Missing	256	171	85	
PFS(day)				0.772
	1085.58 (308.08)	1087.74 (308.12)	1080.22 (308.71)	
Missing	267	181	86	

### Construction of the sarcopenia diagnostic nomogram model

**Fig. 1:**
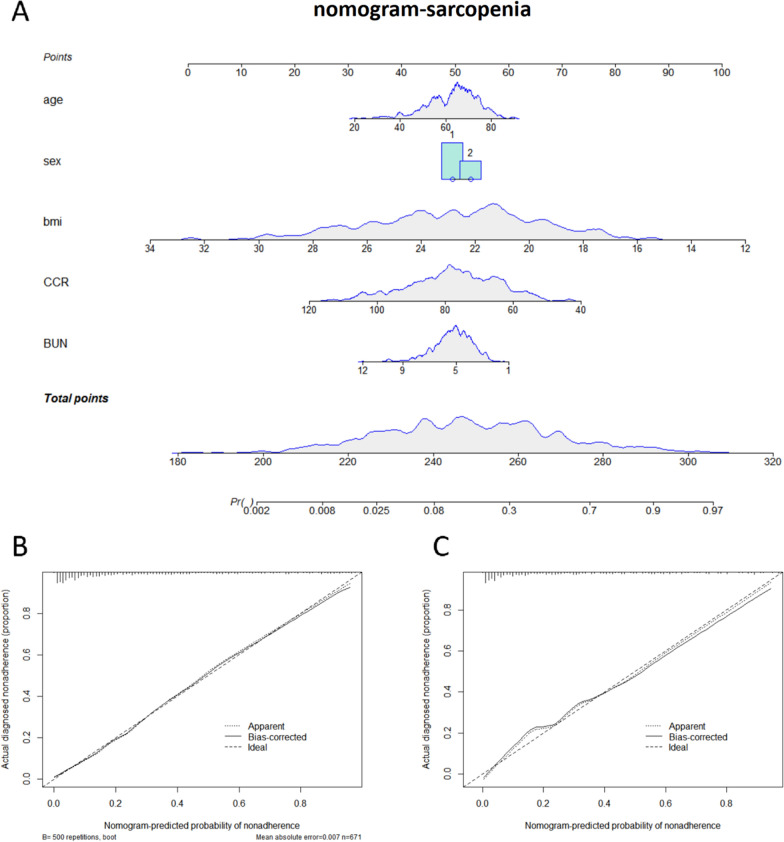
Nomogram Model and Calibration Curve for Sarcopenia Diagnosis. **A** Nomogram model for predicting the risk of sarcopenia in patients. **B** Calibration curve for the training set of the nomogram model. **C** Calibration curve for the validation set of the nomogram model

Based on the results of logistic regression analysis, factors with minor impact were excluded. Age, sex, BMI, CCR, and BUN were used as sarcopenia prediction variables to construct the diagnostic nomogram model in the training set, and a calibration curve was plotted using the validation set. The calibration curve showed that the predicted results were highly similar to the actual results. By calculating the total score of the prediction factors in the nomogram, the risk level of sarcopenia in the target population can be predicted (Fig. 1).

### Construction of the myosteatosis diagnostic nomogram model

**Fig. 2:**
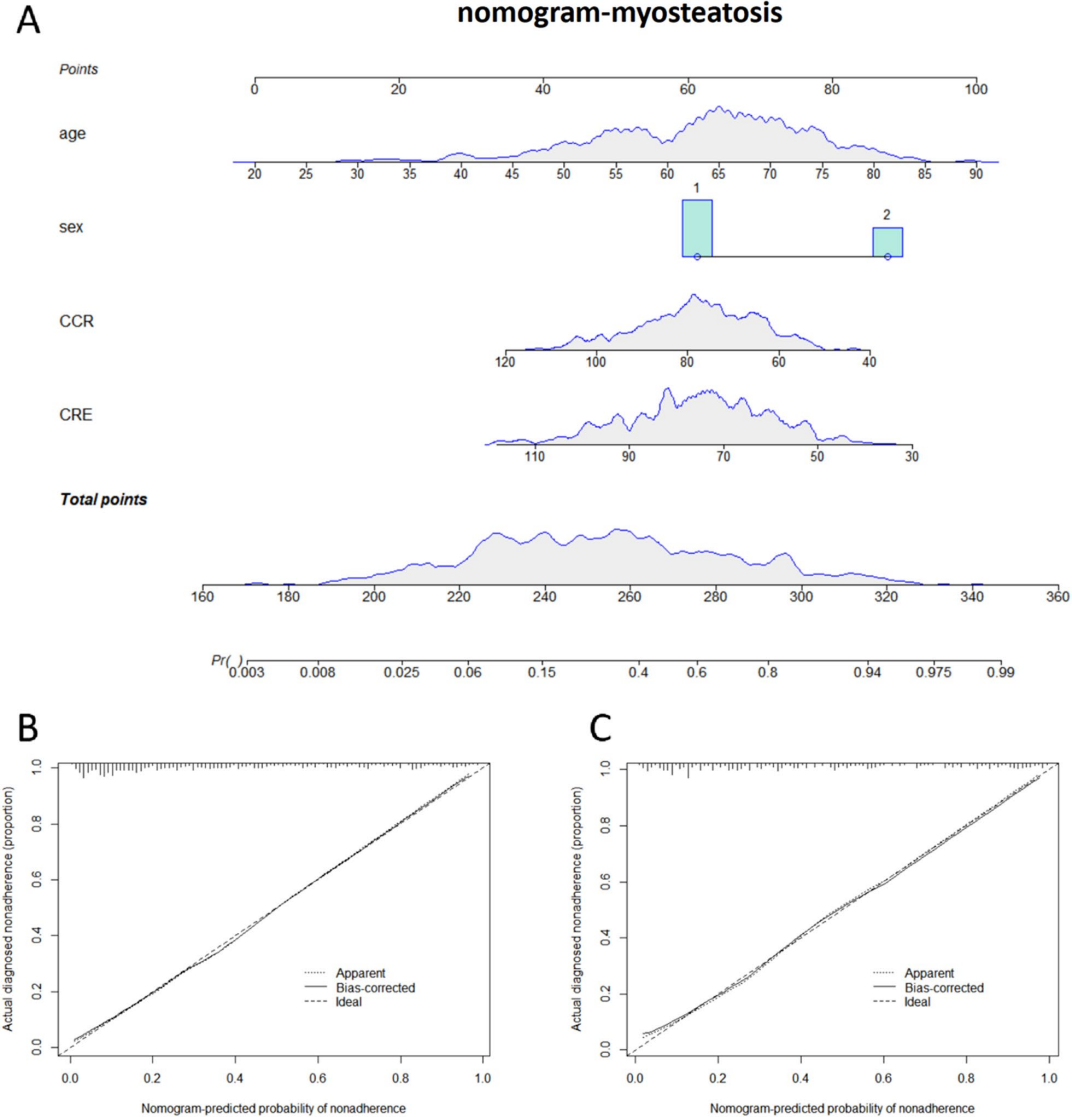
Diagnostic nomogram model and calibration curves for myosteatosis. **A** Nomogram for predicting the risk of myosteatosis in patients. **B** Calibration curve for the training set nomogram. **C** Calibration curve for the validation set nomogram

Based on the results of logistic regression analysis, factors with minor impact were excluded. Age, sex, CCR, and CRE were used as myosteatosis prediction variables. The diagnostic nomogram model was constructed using the training set and validated using the validation set. The calibration curve showed that the predicted results were highly similar to the actual results. By calculating the total score of the prediction factors in the nomogram, the risk level of myosteatosis in the target population can be predicted (Fig. 2).

### Evaluation of predictive ability and clinical efficacy

**Fig. 3:**
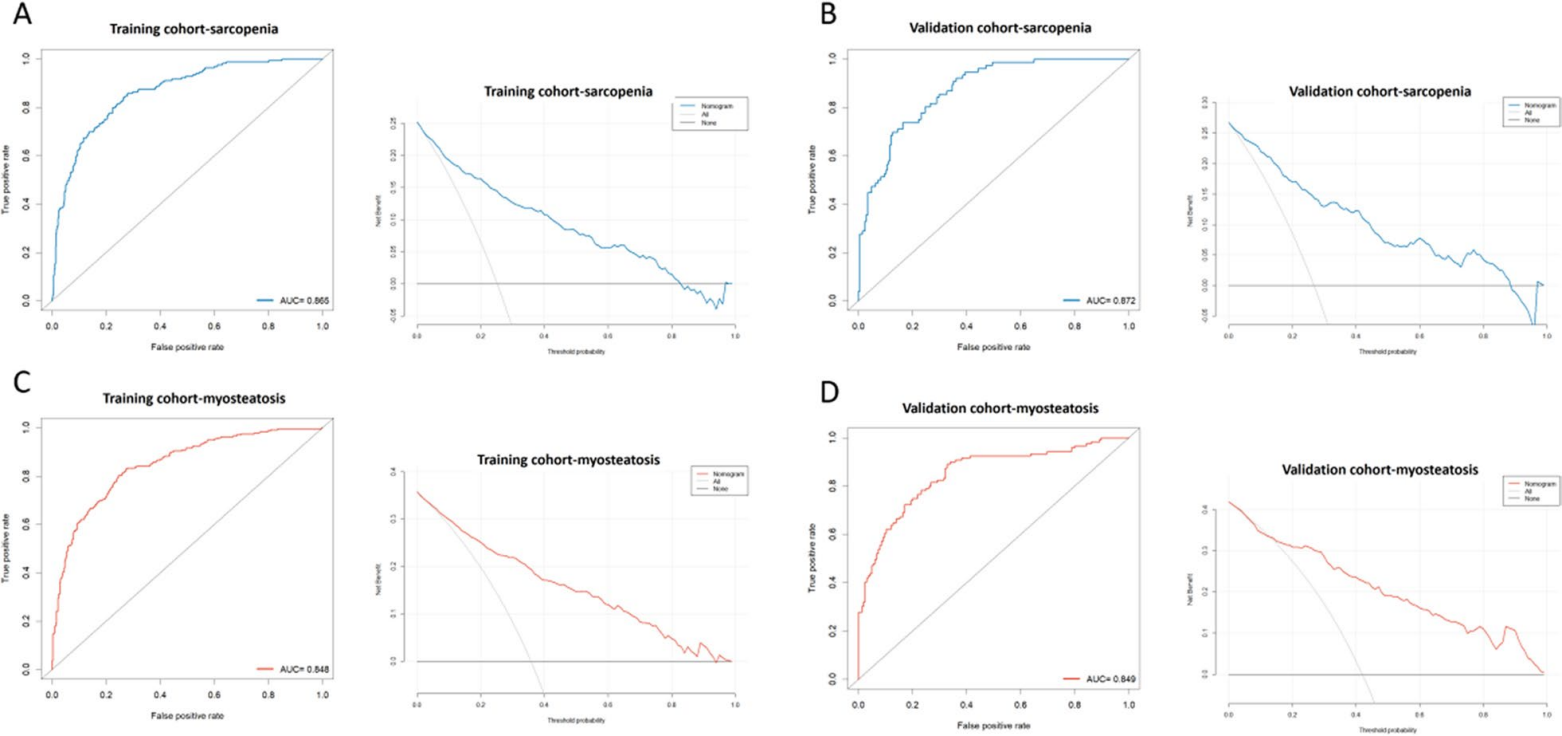
Predictive performance and clinical decision evaluation of nomograms. Nomogram for Sarcopenia: **A** ROC curves and DCA in training cohort, **B** ROC curves and DCA in validation cohort. Nomogram for Myosteatosis: **C** ROC curves and DCA in training cohort, **D** ROC curves and DCA in validation cohort

We evaluated the diagnostic ability of the models using the area under the ROC curve. The results showed that the sarcopenia prediction model (training group AUC: 0.865, validation group AUC: 0.872) had excellent predictive ability. The myosteatosis prediction model (training group AUC: 0.848, validation group AUC: 0.849) also had excellent predictive ability (Fig. 3). In addition, the decision curve showed that both the sarcopenia and myosteatosis diagnostic models had good clinical decision-making ability (Fig. 3).

### Correlation between CCR and imaging parameters

**Fig. 4:**
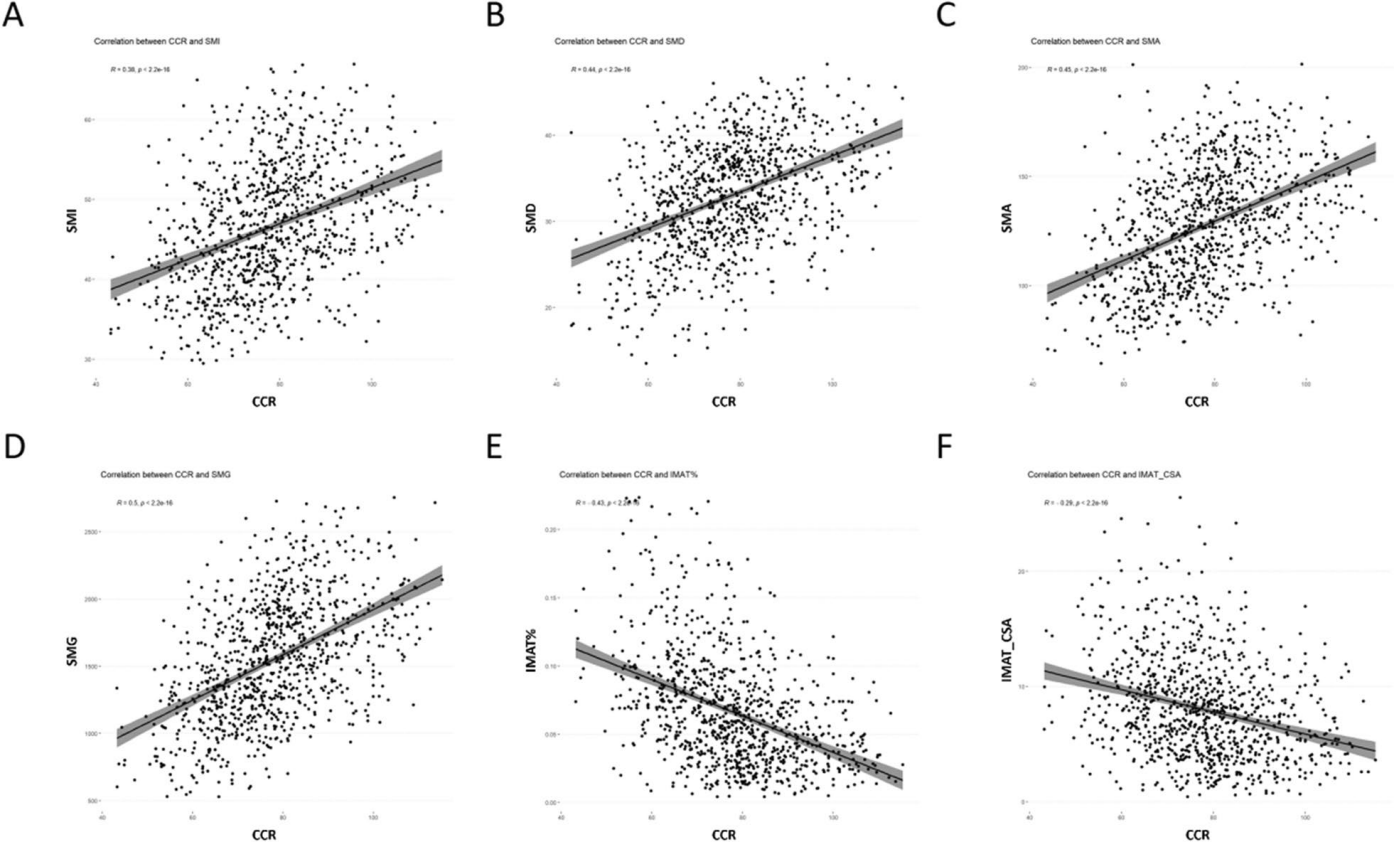
Correlation analysis between CCR and imaging parameters of muscle adipose tissue. **A** Correlation between CCR and SMI (r = 0.38, p < 0.001). **B** Correlation between CCR and SMD (r = 0.44, p < 0.001). **C** Correlation between CCR and SMA (r = 0.45, p < 0.001). **D** Correlation between CCR and SMG (r = 0.50, p < 0.001). **E** Correlation between CCR and IMAT% (r = − 0.43, p < 0.001). **F** Correlation between CCR and IMAT (r = − 0.29, p < 0.001)

We found that CCR was a key predictive factor, and therefore we explored its relationship with relevant imaging indicators. Figure 4 shows the correlation between CCR and radiological measurements. The results indicated that CCR was positively correlated with SMI (r = 0.38, p < 0.001), SMD (r = 0.44, p < 0.001), SMA (r = 0.45, p < 0.001), and SMG (r = 0.50, p < 0.001), while it was negatively correlated with IMAT _CSA(r = − 0.29, p < 0.001) and IMAT% (r = − 0.43, p < 0.001) (Fig. 4).

### Evaluation of the therapeutic effect of sarcopenia and myosteatosis using CCR

**Fig. 5:**
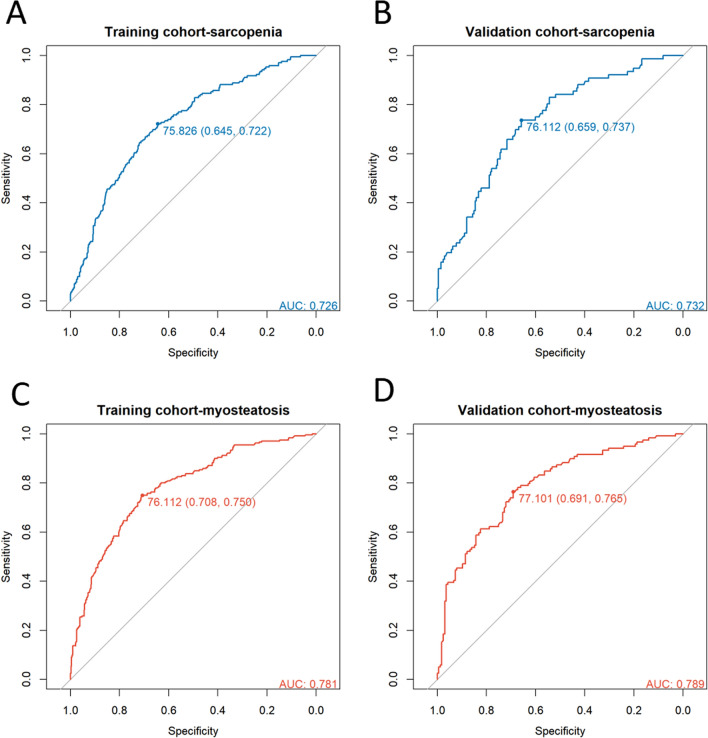
ROC curves and cutoff values of CCR for assessing sarcopenia and myosteatosis. **A**, **B** ROC curves and cutoff values of CCR for assessing sarcopenia in the training and validation sets. **C**, **D** ROC curves and cutoff values of CCR for assessing myosteatosis in the training and validation sets

The results indicated that CCR could be used to evaluate the reduction of skeletal muscle mass (training group AUC: 0.726, validation group AUC: 0.732). CCR showed good independent predictive ability in distinguishing muscle fat infiltration (training group AUC: 0.781, validation group AUC: 0.789). In the training group, the optimal threshold value of CCR for assessing the reduction of skeletal muscle mass was 75.898 (sensitivity = 0.651, specificity = 0.718), and the optimal threshold value of CCR for evaluating muscle fat infiltration was 76.142 (sensitivity = 0.711, specificity = 0.733). Similarly, good results were obtained in the validation group (Fig. 5).

### Evaluation of cachexia using CCR

**Fig. 6:**
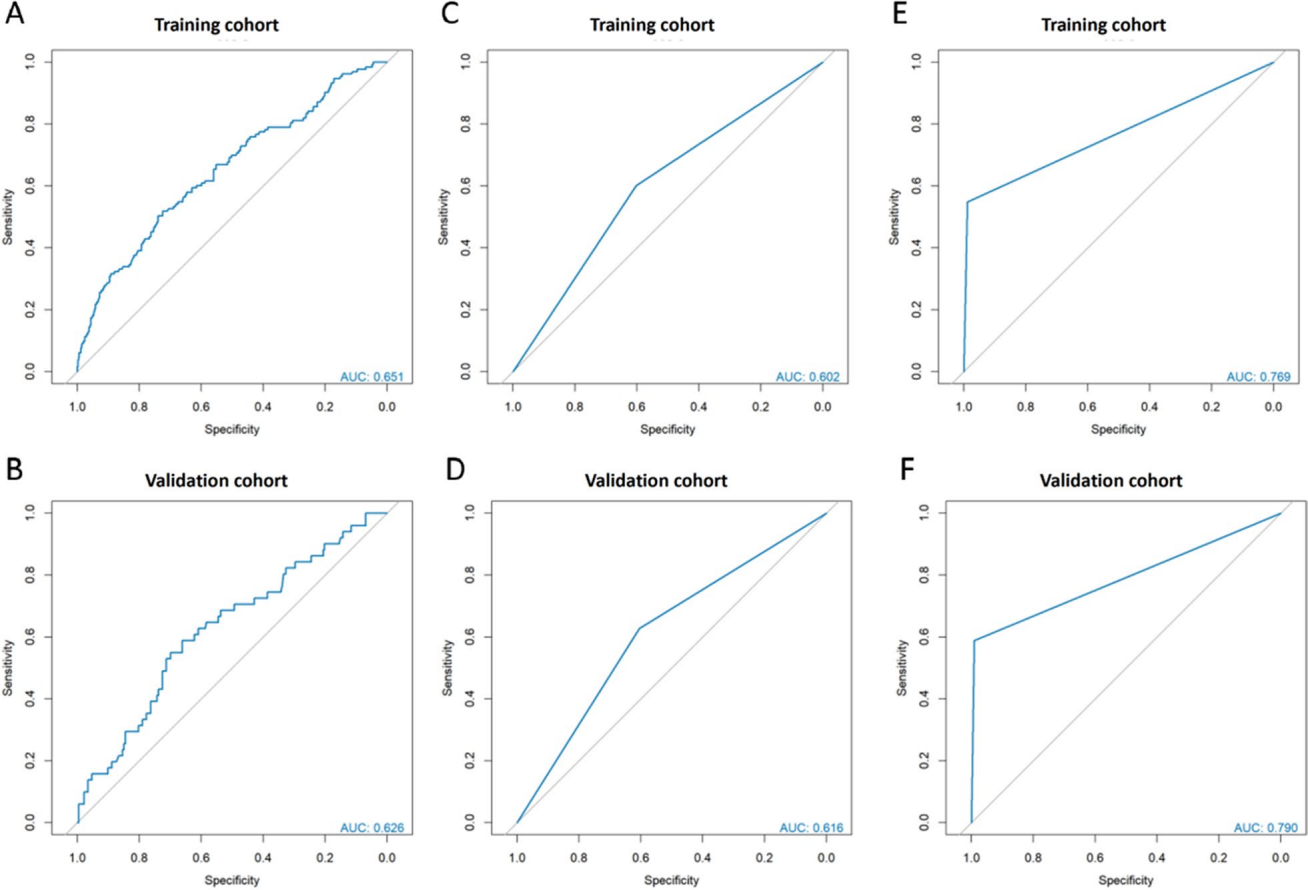
ROC curves for evaluating cachexia using CCR. **A**, **B** ROC curves and cut-off values for evaluating cachexia using CCR in the training and validation groups. **C**, **D** ROC curves and cut-off values for evaluating cachexia using low/high CCR in the training and validation groups. **E**, **F** ROC curves and cut-off values for evaluating cachexia using low CCR and weight loss greater than 0.02 in the training and validation groups

Sarcopenia and muscle fat infiltration are among the characteristics of cachexia patients. We further attempted to use CCR to evaluate cachexia. We evaluated cachexia in the training and validation groups using CCR (training group AUC: 0.651, validation group AUC: 0.626). Cachexia was evaluated when patients were divided into two groups using the cut-off value of CCR (training group AUC: 0.603, validation group AUC: 0.616). When cachexia was evaluated in patients with both low CCR and weight loss greater than 0.02 (training group AUC: 0.769, validation group AUC: 0.790) (Fig. 6).

### Evaluation of clinical prognosis of patients using CCR cut-off value

**Fig. 7:**
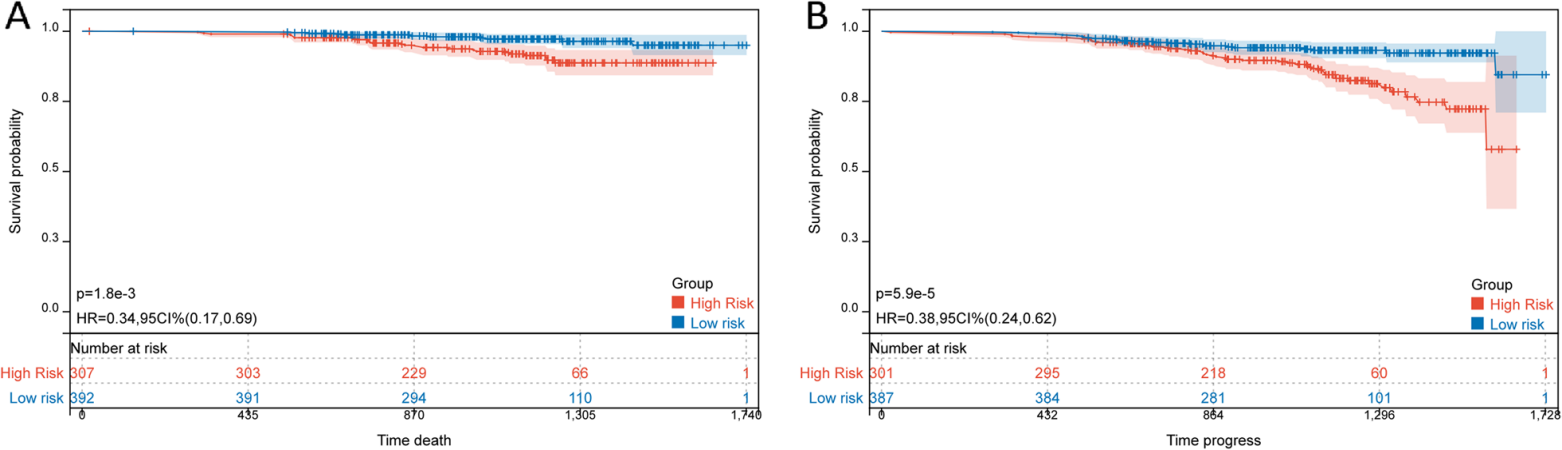
CCR evaluation of clinical prognosis in patients. **A** Evaluation of overall survival (OS) in patients using CCR. **B** Evaluation of disease-free survival (DFS) in patients using CCR

We used the CCR cut-off value to evaluate the clinical prognosis of the overall sample. The results showed that CCR effectively divided patients into high-risk and low-risk groups, assessing the risks of OS and DFS (P < 0.05) (Fig. 7).

## Discussion

Clinical prediction models are used to predict disease risk based on easily obtainable predictors [18]. This study identified risk factors for sarcopenia and myosteatosis by analyzing clinical data and constructed two nomogram-based risk prediction models. We constructed the nomograms using simple clinical variables and successfully predicted disease risk in patients. Therefore, this prediction model has higher practicality and can be used not only in patients with gastrointestinal cancer but also in other populations.

As a non-invasive tool, the nomogram will be a convenient application for clinical doctors [19]. The nomogram prediction models demonstrate the impact of different predictors on the study population [20]. Age, sex, BMI, CCR, and BUN were used to evaluate sarcopenia in our constructed nomogram, while age, sex, CCR, and CRE were used to evaluate myosteatosis. The AUC indicated excellent predictive performance of our nomogram, and calibration curves showed that the predicted probabilities were close to actual outcomes. DCA analysis showed that the nomogram had excellent clinical decision-making ability, which needs to be further validated in clinical practice.

In this study, we found that CCR was a key factor in evaluating sarcopenia and myosteatosis. Further analysis revealed a significant positive correlation between CCR and SMI, and a negative correlation with IMAT%. Furthermore, we confirmed that CCR could be used to assess reductions in skeletal muscle mass and myosteatosis, and reported its diagnostic cutoff value. However, this result needs further validation in different populations. In clinical practice, CCR can be obtained through a simple blood test, without the need for complex imaging or bioelectrical impedance methods, making it more universally applicable.

Diagnosing cachexia in clinical practice is a challenge [21]. In this study, we attempted to use CCR to evaluate cachexia and found that CCR was an effective tool for its diagnosis. When CCR was combined with a weight loss of 2%, its diagnostic ability was significantly improved. Therefore, CCR may become one of the indicators for evaluating cachexia as a substitute for SMI. Meanwhile, the survival curve showed that CCR could effectively stratify patients into high-risk and low-risk groups, evaluating patients' OS and DFS risks, indicating its potential value in clinical prognostic assessment.

Imaging tests have multiple limitations in the routine diagnosis of malnutrition, including the analysis of images obtained in medical records, additional radiation exposure, and high costs [22]. CCR as a serum biomarker, is easily obtained and has a low cost in clinical settings. This study has demonstrated that CCR has the potential to function as a biomarker for diagnosing muscle mass and myosteatosis.This could aid in early disease detection and enable timely interventions, offering the advantages of being non-invasive and cost-effective. In addition, this study constructed a prediction model based on serum indicators and basic patient characteristics, without using difficult-to-obtain indicators. This model serves as a valuable tool for clinicians and nutritionists to conduct risk assessments, facilitating the timely identification of conditions that require early interventions and personalized treatment strategies, ultimately leading to improved patient outcomes. Additionally, our research findings are adaptable and practical for implementation in a variety of healthcare settings, including community hospitals.

However, this study still has several limitations. Firstly, further prospective collection and analysis are needed to investigate the relationship between CCR and other skeletal muscle mass indicators, such as walking speed and grip strength. Secondly, this research did not compare the data with other variables, including functional status, pathological outcomes, or other biomarker assessments, such as the Glasgow prognostic score and specific gut microbiota [23]. Thirdly, the findings are based on data from a single center, necessitating further validation to determine if the results can be generalized to other ethnic groups or patients with different diseases. Lastly, the evaluation effectiveness of the model developed in this study requires additional external validation.

## Conclusion

In this study, we established a nomogram to evaluate the risk of sarcopenia and myosteatosis in clinical practice. The model demonstrated high accuracy and can assist clinicians and nutritionists in making assessments. Additionally, we found the potential of CCR in evaluating sarcopenia, myosteatosis, and cachexia. These findings may be helpful for the early diagnosis of muscle and fat depletion and could improve the management and prognosis of patients.

### Supplementary Information

**Additional file 1: Table S1.** Logistic regression analysis results of risk factors for sarcopenia.

**Additional file 2: Table S2.** Logistic regression analysis results of risk factors for myosteatosis.

## Data Availability

All relevant data and materials have been involved in the article. Further inquiries can be directed to the corresponding authors.
