# Supplementary material for: Sarcopenia and myosteatosis diagnostic tool for gastrointestinal cancer: creatinine to cystatin C ratio as evaluation marker
Source: J Transl Med. 2023 Oct 20;21:744. doi: 10.1186/s12967-023-04628-z (PMC10589997; doi:10.1186/s12967-023-04628-z)
Supplement: Supplementary file 1 — Additional file 1: Table S1. Logistic regression analysis results of risk factors for sarcopenia. [file 12967_2023_4628_MOESM1_ESM.docx]

| **Table S1. Logistic regression analysis results of risk factors for sarcopenia** | | | | |
| --- | --- | --- | --- | --- |
|  | **[univariate analysis](javascript:;)** | | **[multivariate analysis](javascript:;)** | |
| **Characteristic** | **HR(95%CI)** | **P-value** | **HR(95%CI)** | **P-value** |
| Age(year) | 1.042(1.024-1.061) | <0.001 | 1.056(1.029-1.085) | <0.001 |
| Sex(n) | 2.244(1.569-3.213) | <0.001 | 3.291(1.541-7.157) | 0.002 |
| Weight(kg) | 0.905(0.885-0.925) | <0.001 | 1.289(0.747-2.193) | 0.355 |
| Height(m) | 0.312(0.035-2.786) | 0.296 |  |  |
| BMI(kg/m^2) | 0.645(0.591-0.7) | <0.001 | 0.386(0.229-0.645) | <0.001 |
| BSA | 0.007(0.002-0.023) | <0.001 | 0.03(0-6.921) | 0.649 |
| Location(n) | 1.413(0.996-2.013) | 0.054 |  |  |
| Ratio of body weight loss | 1.062(1.025-1.101) | <0.001 | 0.932(0.845-1.026) | 0.143 |
| CCR(umol/mg) | 0.933(0.917-0.948) | <0.001 | 0.949(0.923-0.975) | <0.001 |
| Cre(umol/L) | 0.958(0.945-0.971) | <0.001 | 0.986(0.961-1.012) | 0.287 |
| CysC(mg/L) | 1.776(0.614-5.105) | 0.287 |  |  |
| TNF(ng/ml) | 1.005(0.998-1.013) | 0.166 |  |  |
| CRP(mg/L) | 1.017(1.007-1.029) | 0.002 | 1.01(0.997-1.025) | 0.132 |
| BUN(mmol/L) | 0.826(0.726-0.935) | 0.003 | 0.806(0.682-0.947) | 0.01 |
| GLU(mmol/L) | 0.906(0.783-1.029) | 0.154 |  |  |
| ALB(g/L) | 0.885(0.847-0.922) | <0.001 | 1.006(0.94-1.077) | 0.868 |
| NEUT(x10^9/L) | 1.03(0.926-1.139) | 0.555 |  |  |
| LYMPH(x10^9/L) | 0.807(0.585-1.104) | 0.186 |  |  |
| HGB(x10^12/L) | 0.981(0.974-0.989) | <0.001 | 0.991(0.978-1.003) | 0.133 |
| WBC(x10^9/L) | 1.012(0.923-1.101) | 0.787 |  |  |
| PLT(x10^9/L) | 1.001(0.998-1.003) | 0.623 |  |  |
