# Supplementary material for: Sarcopenia and myosteatosis diagnostic tool for gastrointestinal cancer: creatinine to cystatin C ratio as evaluation marker
Source: J Transl Med. 2023 Oct 20;21:744. doi: 10.1186/s12967-023-04628-z (PMC10589997; doi:10.1186/s12967-023-04628-z)
Supplement: Supplementary file 2 — Additional file 2: Table S2. Logistic regression analysis results of risk factors for myosteatosis. [file 12967_2023_4628_MOESM2_ESM.docx]

| **Table S2. Logistic regression analysis results of risk factors for myosteatosis** | | | | |
| --- | --- | --- | --- | --- |
|  | **[univariate analysis](javascript:;)** | | **[multivariate analysis](javascript:;)** | |
| **Characteristic** | **HR(95%CI)** | **P-value** | **HR(95%CI)** | **P-value** |
| Age(year) | 1.059(1.042-1.078) | <0.001 | 1.095(1.068-1.125) | <0.001 |
| Sex(n) | 6.496(4.58-9.288) | <0.001 | 6.088(3.117-12.174) | <0.001 |
| Weight(kg) | 0.973(0.958-0.987) | <0.001 | 0.857(0.571-1.269) | 0.446 |
| Height(m) | 0(0-0.001) | <0.001 | 0(0-516.695) | 0.247 |
| BMI(kg/m^2) | 1.026(0.977-1.077) | 0.309 |  |  |
| BSA | 0.09(0.034-0.232) | <0.001 | 2.718(0-3.191) | 0.321 |
| Location(n) | 1.391(1.014-1.913) | 0.041 | 1.058(0.699-1.6) | 0.79 |
| Ratio of body weight loss | 1.009(0.974-1.044) | 0.618 |  |  |
| CCR(umol/mg) | 0.91(0.893-0.925) | <0.001 | 0.96(0.937-0.983) | <0.001 |
| Cre(umol/L) | 0.934(0.921-0.947) | <0.001 | 0.961(0.939-0.982) | <0.001 |
| CysC(mg/L) | 0.824(0.312-2.16) | 0.695 |  |  |
| TNF | 1.003(0.996-1.01) | 0.352 |  |  |
| CRP(mg/L) | 1.017(1.006-1.03) | 0.004 | 1.013(0.999-1.028) | 0.086 |
| BUN(mmol/L) | 0.88(0.787-0.982) | 0.023 | 0.941(0.809-1.092) | 0.423 |
| GLU(mmol/L) | 1.075(0.967-1.194) | 0.175 |  |  |
| ALB(g/L) | 0.951(0.916-0.987) | 0.008 | 1.012(0.949-1.079) | 0.725 |
| NEUT(x10^9/L) | 1.034(0.94-1.14) | 0.478 |  |  |
| LYMPH(x10^9/L) | 0.758(0.566-1.007) | 0.059 |  |  |
| HGB(x10^12/L) | 0.983(0.977-0.99) | <0.001 | 0.991(0.98-1.002) | 0.115 |
| WBC(x10^9/L) | 0.996(0.916-1.078) | 0.929 |  |  |
| PLT(x10^9/L) | 1(0.998-1.002) | 0.747 |  |  |
